# Simulated subacromial injection instruction improves accuracy and skill level: a model for musculoskeletal procedural training

**DOI:** 10.1186/s12909-024-05456-5

**Published:** 2024-05-14

**Authors:** Rishi Chatterji, Jake Foote, Mike Fry, Ashley Erwin, Joe Crutcher, William Kesto

**Affiliations:** 1 Department of Orthopedic Surgery, Ascension Providence Hospital – MSU, 16001 W. Nine Mile Rd., 4th Floor Fisher Building, Rm. #405, Southfield, MI 48075 USA; 2 Department of Family Medicine, Ascension Providence Hospital – MSU, 16001 W. Nine Mile Rd., 4th Floor Fisher Building, Rm. #405, Southfield, MI 48075 USA; 3 Simulation and Education Center Van Eslander Surgical Innovation Center, Ascension Providence Hospital – MSU, Novi, MI USA; 4 Ascension Providence Hospital – MSU, 16001 W. Nine Mile Rd., 4th Floor Fisher Building, Rm. #405, Southfield, MI 48075 USA; 5 The Core Institute, Novi, MI USA

**Keywords:** Subacromial injection, Education, Bioskills simulation, Shoulder, Cadaveric study

## Abstract

**Background:**

Musculoskeletal (MSK) complaints often present initially to primary care physicians; however, physicians may lack appropriate instruction in MSK procedures. Diagnostic and therapeutic injections are useful orthopedic tools, but inaccuracy leads to unnecessary costs and inadequate treatment. The authors hypothesized that trainees afforded the opportunity to practice on a cadaver versus those receiving visual-aided instruction on subacromial injections (SAI) will demonstrate differences in accuracy and technique.

**Methods:**

During Spring of the year 2022, 24 Internal Medicine and Family Medicine residents were randomly divided into control and intervention groups to participate in this interventional randomized cadaveric study. Each group received SAI instruction via lecture and video; the intervention group practiced on cadavers under mentored guidance. Subjects underwent a simulated patient encounter culminating in injection of latex dye into a cadaveric shoulder. Participants were evaluated based on a technique rubric, and accuracy of injections was assessed via cadaver dissection.

**Results:**

Twenty-three of twenty-four participants had performed at least one MSK injection in practice, while only 2 (8.3%) of participants had performed more than 10 SAIs. There was no difference in technique between control 18.4 ± 3.65 and intervention 19.2 ± 2.33 (*p* = 0.54). Dissections revealed 3 (25.0%) of control versus 8 (66.7%) of intervention injections were within the subacromial space. Chi-Square Analysis revealed that the intervention affected the number of injections that were within the subacromial space, in the tissues bordering the subacromial space, and completely outside the subacromial space and bordering tissues (*p* = 0.03). The intervention group had higher self-confidence in their injection as opposed to controls (*p* = 0.04). Previous SAI experience did not affect accuracy (*p* = 0.76).

**Conclusions:**

Although primary care physicians and surgeons develop experience with MSK procedures in practice, this study demonstrates a role for early integrated instruction and simulation to improve accuracy and confidence. The goal of improving accuracy in MSK procedures amongst all primary care physicians may decrease costs and avoid unnecessary referrals, diagnostic tests, and earlier than desired surgical intervention.

## Background

MSK complaints make up a significant portion of primary care visits. Up to 70% of all new MSK problems are treated by primary care physicians, and 90% of these problems can be definitively managed in this setting [1–3]. Shoulder pain is the third most common MSK complaint and is responsible for roughly 11–27% of all MSK complaints in the primary care setting [4, 5]. Many shoulder concerns can be initially managed with intra-articular glenohumeral or peri-articular subacromial injections to reduce inflammation and provide pain relief [6, 7]. SAIs are often indicated for subdeltoid bursitis, adhesive capsulitis, rotator cuff tendinosis and rotator cuff impingement [8]. The ability of primary care physicians to manage these issues saves time for the patient, is cost-effective, prevents unnecessary referrals, and improves care. However, this requires a unique skill set that many primary care physicians may lack or feel comfortable performing accurately.

Injections can serve as both a diagnostic and therapeutic tool for a variety of MSK pathologies. However, correct placement of these injections is essential for their success. Inaccurate injections may lead to inaccurate diagnoses, poor clinical outcomes, infection, and earlier than anticipated need for surgery [7, 8]. Moreover, accurate injections isolated to the appropriate location have demonstrated better pain and functional scores in patients compared with injections that are only partially accurate [7]. Although MSK complaints often first present in the primary care setting, primary care physicians may not feel comfortable managing these issues due to inadequate exposure to basic MSK procedures during residency [9]. Also, residents who previously possessed sufficient comfort and ability at one time may suffer from declining skills due to lack of appropriate training and reinforcement [3, 9].

Accuracy of SAIs has been previously studied in cadaveric models, but to our knowledge there have been no studies to date that observe the effect of formal instruction and simulation training on SAI accuracy. The objectives of this study were to evaluate the accuracy of SAIs performed by Internal Medicine and Family Medicine physicians, and to assess whether simulated training on cadavers improved injection accuracy compared to traditional visual-aided and lecture-format learning. The investigators also sought to determine if a correlation exists between injection accuracy and prior injection experience. We hypothesized that trainees given the opportunity to practice on a cadaveric specimen would perform better than those who solely received verbal and visually-aided instructions. Furthermore, we anticipated that trainees with prior injection experience would achieve higher rates of accuracy with SAIs compared to those without experience, regardless of intervention.

## Methods

Prior to data collection, the protocol was reviewed by the appropriate Research Committee and Institutional Review Board for the primary institution. The protocol was deemed exempt with minimal risk by the Ascension Providence Hospital Institutional Review Board on March 22, 2021 as IRB Study # 1709105-1. All procedures were performed in accordance with ethical guidelines for Human Subjects Research. Primary care physicians, including both residents and attending physicians from the Internal Medicine and Family Medicine departments were recruited to participate in the study from January to April, 2022. Study size was chosen such there was sufficient representation from both departments, while limiting injections to two per cadaver. All subjects provided consent to participate in the study. Twelve Cadaveric shoulder specimens were obtained from Anatomy Gifts Registry (Hanover, MD) and permission was obtained for use as well as photography and videography.

All participants were recruited from the family medicine and internal medicine departments via email from the respective program directors. Each participant subsequently completed a brief survey to assess prior experience, confidence level, and proficiency with injections and additional MSK procedures prior to this study. Subjects were provided with opaque envelopes with an assigned identification number and randomly assigned to one of two groups (intervention and control). The participants were told that they were receiving instruction on SAI and would be evaluated for accuracy and technique. However, all participants were not aware that there were two different training methods, as they were evaluated individually, trained in different rooms, and unable to discuss their experience until after the completion of the study session.

A curriculum was developed to teach, test, and evaluate the skill and proficiency of the SAI procedure. The design was based on a previous study that used a combination of lectures, practice sessions, and expert advice, with the goal of understanding appropriate indications and sterile technique [10]. All participants received basic education on the background, indications, technique, and outcomes for SAIs. This consisted of a twenty-minute lecture, followed by a four-minute video demonstrating appropriate sterile injection technique. Overall there was an educator to learner ratio of 1:3 for each session.

**Fig. 1:**
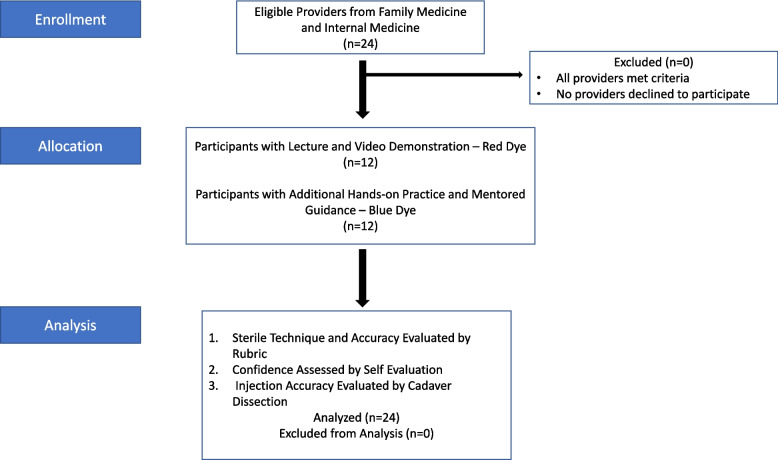
Flow diagram demonstrating how groups were randomly separated by learning style. They were then evaluated by the same method, which included a scoring rubric as well as cadaver dissection for accuracy analysis

Participants in the control group then went straight to testing. Those in the intervention group were provided an additional ten minutes of mentored guidance to practice the SAI on a cadaver (Fig. 1). This consisted of practicing sterile preparation and performing a SAI using a 22-gauge needle with 10cc of normal saline on a cadaveric shoulder under the guidance of an orthopedic surgery resident proficient in injections as taught by an orthopedic sports medicine specialist. Participants were given feedback and allowed to ask questions during this practice session.

**Fig. 2:**
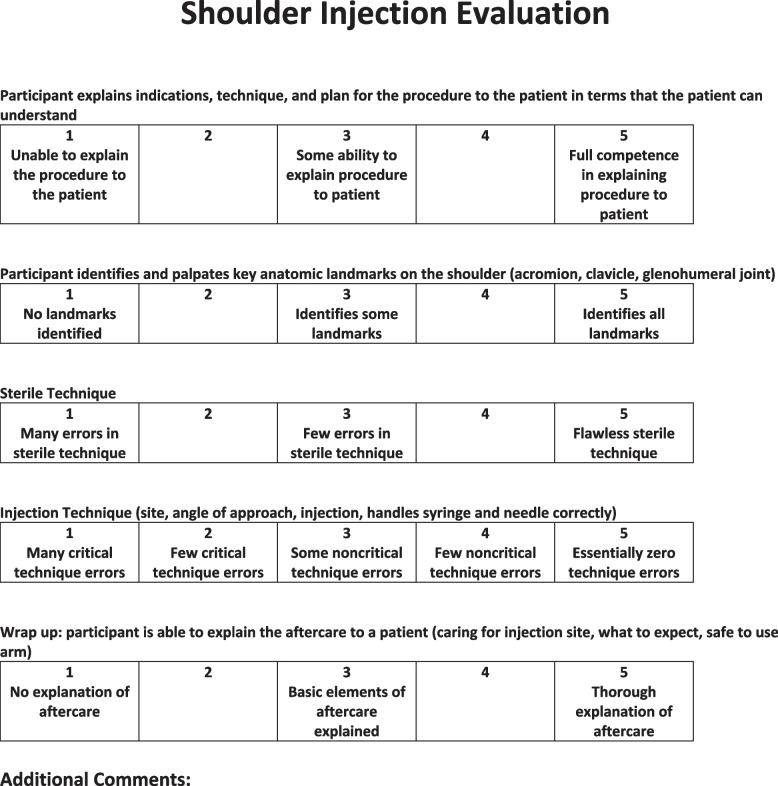
Technique Rubric used to evaluate each individual during the injection process. Each individual was assessed by a sports fellowship-trained orthopedic surgeon, and the total score was from 1–25

Performance evaluation was based on a previously described method [11]. Testing was conducted one participant at a time and evaluated by an orthopedic surgery attending with sports fellowship training using a scoring rubric. Participants performed a SAI using 10cc of colored latex dye (HX-Injection Medium, Holden’s Medium Latex; Macungie, PA) into a cadaveric shoulder positioned upright in a clamp. The control group injected red dye, while the intervention group injected blue dye. Participants were not told that there were different colored dyes for injection. The orthopedic surgeon used the pre-designed scoring form (Fig. 2) to consistently assess sterile technique and injection accuracy on a total scale of 1–25. After testing, participants completed another survey that inquired about their confidence in their injection after training, as well as their overall experience.

Accuracy of injection was evaluated one week later. Two orthopedic surgery residents previously trained thoroughly in both subacromial dissections and various surgical approaches to the shoulder dissected each cadaveric shoulder via an extensile deltoid split approach. The subacromial space was defined as being bordered superiorly by the acromion and coracoacromial ligament, inferiorly by the supraspinatus and humeral head, medially by the supraspinatus fascia, laterally by the subdeltoid fascia in line with the lateral border of the acromion, and posteriorly by the infraspinatus and subdeltoid fascia [12, 13]. The colored dye was located, and each injection was graded as either within the subacromial space and bordering tissues (accurate), in tissues only directly bordering the space (inaccurate), or completely outside the desired injection area (complete miss). These areas were determined based on the understanding that an injection completely within the subacromial space would serve as an accurate injection, while an injection involving the bordering tissues would indicate that most of the medicine did not enter the desired location, but it is possible that in a clinical scenario this may partially diffuse into the subacromial space given the proximity. A complete miss would indicate no medicine in or near the desired location. The final location of dye was also confirmed by the sports fellowship-trained orthopedic surgeon.

### Statistical analysis

Microsoft Excel was used for all statistical analysis. Two-tailed T tests were used to compare average evaluation score and post-testing confidence level between intervention and control groups. Two-Tailed Fisher’s Exact Test was used to compare injection accuracy of injections within the subacromial space with or without bordering tissue involvement vs. injections completely outside the subacromial space and bordering tissues between intervention and control. Statistical significance was defined as *p* < 0.05.

## Results

Twenty-four primary care physicians were available for the study, consisting of twelve internal medicine and twelve family medicine physicians. All subjects completed the full training session for the group they were assigned, as well as the pre- and post-testing evaluations.

Training level was broken down by Post-Graduate Year (PGY) with 8 out of 24 (33.3%) PGY-1 participants, 9 out of 24 (37.5%) PGY-2 participants, 2 out of 24 (8.33%) PGY-3 participants, and 5 out of 24 (20.8%) past PGY-3 participants. Subjects had varying degrees of previous experience with MSK procedures. 23 out of 24 (95.8%) participants had performed at least one MSK injection in practice, while 12 out of 24 (50%) participants had performed at least one SAI. Of those with previous experience of MSK injection, 90.5% had experience with knee injection, followed by SAIs (47.6%), glenohumeral injections (19.0%), and greater trochanteric bursitis injections (4.8%). Only 2 subjects (8.3%) had performed more than 10 SAIs prior to the study (Table 1). Overall mean technique score based on the orthopedic surgeon’s evaluation was 18.8 out of 25. No difference was found between control (18.4 ± 3.65) and intervention (19.2 ± 2.33) groups (*p* = 0.54).

**Table 1:** Participant demographics from 24 participants: previous experience and type of physician

Demographic Data (*n* = 24)	#	%
Type of Physician		
Family Medicine	12	50%
Internal Medicine	12	50%
Attending Physician	5	26.3%
Resident	19	79.2%
Previous MSK Rotation?		
Yes	11 (R = 6, B = 5)	45.8%
No	13 (R = 6, B = 7)	54.2%
# of Previous SAIs Performed		
0	12 (R = 6, B = 6)	50.00%
1–5	5 (R = 2, B = 3)	20.83%
6–10	5 (R = 1, B = 3)	20.83%
11–20	1 (R = 2, B = 0)	4.17%
> 20	1 (R = 1, B = 0)	4.17%

Demographic data of participants in the study. This demonstrates the overall breakdown as well as the previous experience of each participant with MSK procedures and the SAI specifically. In the survey, a previous MSK rotation was defined as at least 1 month rotation during residency that involved diagnosing and treating knee, hip, and shoulder MSK pathology

R = Red Control Group; B = Blue intervention control group

**Fig. 3:**
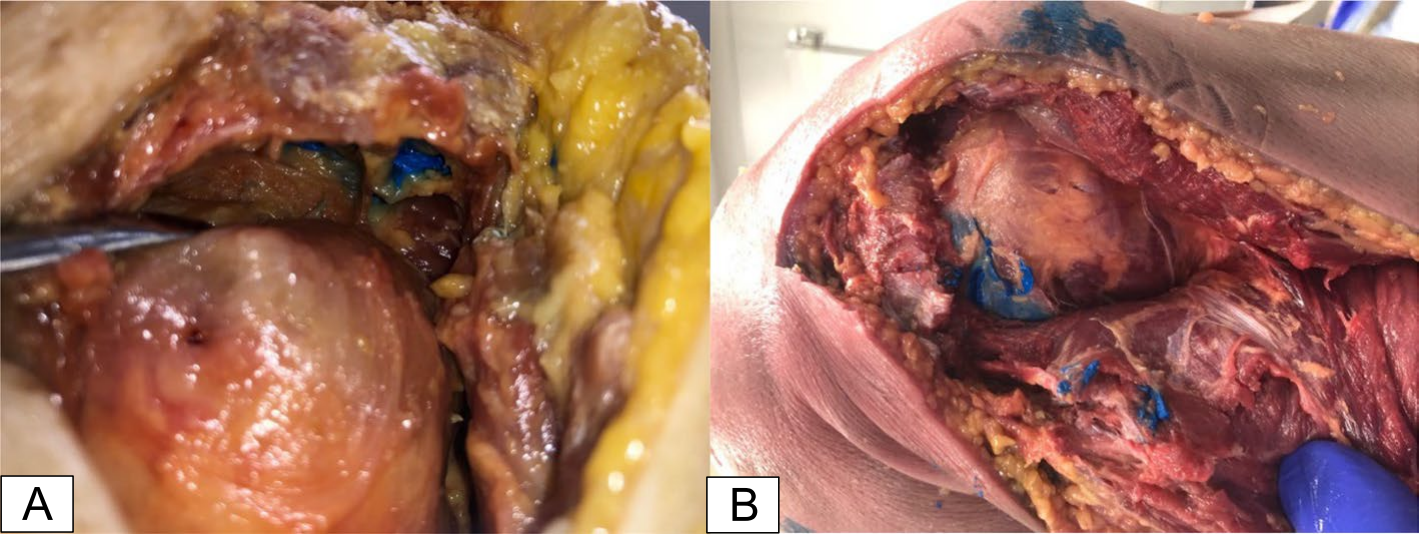
**A** Cadaver dissection demonstrating blue dye successfully within the subacromial space. **B** Cadaver dissection demonstrating blue dye within the subacromial space as well as along the tissues bordering the subacromial space including the supraspinatus and infraspinatus

Twelve cadaveric shoulders in total were used so that there was one blue injection and one red injection in each shoulder. Cadaver dissection for analysis of injection accuracy revealed that 11 of 24 (45.8%) participants demonstrated an accurate injection (Fig. 3A), 9 of 24 (37.5%) participants demonstrated an inaccurate injection within only the bordering tissues (Fig. 3B), while 13 of 24 (54.7%) participants were completely outside the subacromial space (complete miss).

A significant difference in accuracy was seen between groups, with accurate injections attained by 66.7% of those in the intervention group compared to 25% of control group participants. There was not a clear correlation between training level and accuracy as determined by cadaver dissection, with 40% of attending physicians within the subacromial space and 40% of attending physicians fully outside the subacromial space and bordering tissues versus 47.4% of residents within the subacromial space and 31.6% of residents fully outside the subacromial space. Fisher’s Exact Test revealed that there was a correlation between intervention and location of injection, such that the intervention affected whether or not the injection was within the subacromial space with or without direct bordering tissue involvement versus an injection completely outside of the subacromial space and bordering tissues between the two groups with statistical significance (*p* = 0.02).

Post-testing evaluation of self-confidence level on a scale of 1–5 was found to be significantly higher in the intervention group (3.67) compared to the control group (3.08) with (*p* = 0.04). Notably, 29.2% of overall participants rated their self-confidence as 4 or higher prior to the study, while 58.3% of overall participants rated self-confidence as 4 or higher after the study (Table 2). Analysis of previous experience showed that number of previous SAIs performed did not affect accuracy, regardless of group (*p* = 0.76). In fact, both groups had half participants with experience of 0 SAIs, while the control group had three individuals who had performed more than 11 previous SAIs, while the intervention group did not have any over 11 previous SAIs.

**Table 2:** Accuracy and confidence for control vs. intervention groups

**Injection Evaluation**	**Control (Red)** *n* = *12*	**Intervention (Blue)** *n* = *12*	***P*-Value**
Mean Sterile Technique and Accuracy During Injection (scale of 25)	18.4	19.2	0.54
Mean Confidence in tested injection (scale 1–5)	3.08	3.67	0.04
Cadaver Analysis			
Injection: Accurate	25.0%	66.7%	0.02
Injection: Complete Miss	58.3%	8.33%	0.02

Accuracy and technique evaluation, demonstrating that the intervention of simulated training did lead to increased incidence of an injection completely within the subacromial space as opposed to solely lecture and video-based learning. Confidence also appeared to marginally improve in the intervention group. Accurate injection indicates that the injection was completely within the subacromial space, while complete miss indicates that the injection was not within the subacromial space or the tissues surrounding the subacromial space

## Discussion

This study represents a unique assessment of the role of integrated instruction and simulation in improving self-confidence and accuracy while performing a common MSK procedure. Despite varying levels of previous experience amongst subjects, there was a clear correlation between simulation and expert feedback with increased SAI accuracy. The intervention group was more accurate with injections (66.7% vs. 25%, *p* = 0.02) and more confident post-evaluation (3.67 vs. 3.08, *p* = 0.04) compared to the control group. The overall technique scores were improved in the intervention group; however, this difference was marginal (*p* = 0.54). Most of the comments regarding lower scores involved issues with sterility and post-procedural instructions such as accidentally touching the injection site after sterile prep or failing to describe the indications or post-procedural expectations to the simulated patient. This may indicate that basic understanding and execution of procedural skills may be lacking in addition to accuracy.

Previous studies have examined MSK procedure proficiency amongst physicians in training [3, 10, 11]. However, this study is unique in that objective accuracy, technique, and self-confidence were measured among a variety of primary care physicians while also directly assessing the effects of formal instruction and simulated practice on injection accuracy and overall performance.

SAI was specifically chosen as the procedure to be examined in this study due to the proficiency required to accurately inject this smaller space and common need for these injections vs. the glenohumeral joint. Although both knee and shoulder injections have been assessed in the previous studies, the subacromial space is significantly smaller than the knee, making it one of the more difficult joints to inject. The subacromial space has been found to be between 2.28 and 3.93 cm^3 in volume analyses [14, 15]. This is significantly smaller than the knee joint, which was found to be around 6.7 cm^3 in synovial volume, and larger with effusions present [16]. Furthermore, the subacromial space has been found to be significantly smaller in patients with rotator cuff tears [14, 15]. This is clinically relevant, as many patients receiving SAIs may have evidence of rotator cuff tears.

Relative indications for SAIs may include serving as a diagnostic and therapeutic tool for subacromial impingement, adhesive capsulitis, calcific tendonitis, subdeltoid bursitis, rotator cuff tendinosis, and first line treatment for rotator cuff tears prior to surgery [7, 8]. Although other joints are small in size, such as the wrist, shoulder injections in the primary care setting continue to be one of the most important joints to consider, as shoulder pain is prevalent in up to 7–34% of the population [17]. Additionally, shoulder impingement has been shown as the underlying cause for the majority of shoulder complaints [4, 17], highlighting the importance of SAIs compared to other types of shoulder injections.

Deficiencies in MSK procedural training likely stem from deficiencies in medical school education. Freedman and Bernstein found that 82% of clinicians who had just completed medical school failed a basic MSK competency assessment consisting of multiple questions testing knowledge that had been validated by 124 orthopedic surgery program directors with each question rated and validated on a scale of 1 to 10 [18]. Another study analyzed procedural skills between urban vs rural family medicine residents, including orthopedic skills such as application of casts and splints, glenohumeral and knee injections, as well as shoulder reductions. The study revealed that first year residents from various medical schools initially had similar competency levels. However, self-assessed competence and experience was higher after graduation from a rural program, and it was suggested that this may be due to more training from specialty-specific preceptors since family medicine resident may have a larger role of care in the rural setting [19].

Previous studies have analyzed the ability of residents to perform procedures such as diagnostic and therapeutic injections affecting joints. One study of physical medicine and rehabilitation residents examined 15 injection techniques and found that an instructional course can significantly improve immediate skill and comfort level. Residents scored 59.3% on a multiple choice exam prior to the instructional course with 0% of residents demonstrating proficiency based on assessment by a committee. After the instructional course, the residents scored 90.6% on a multiple choice exam, with 63.6% of residents demonstrating proficiency [10]. Preisner et al. examined knee and shoulder aspiration and injection techniques amongst internal medicine residents. They assessed whether procedural skills learned after an in-depth simulation course were preserved over time with periodic web-based review and practice on models. After 6–30 months, they found that zero residents demonstrated the same level of proficiency that they had shown immediately after the instructional course; however, the group that completed the web-based review performed better on the procedural skills than the group that did not receive review materials [3]. Long-term skill proficiency and comfort level is crucial for procedures to effectively manage patients. A longitudinal study on 56 residents and 8 faculty physicians in a 4-year curriculum including intra-articular injections found that comfort level increased each year and number of procedures performed each year increased. This suggests that long-term proficiency and comfort with procedures can be achieved if there is consistent reinforcement via an integrative clinical setting [9]. We therefore believe that future studies may build upon this investigation by examining whether there is attrition or improvement in skill simply by reviewing the materials over the years versus practice in MSK procedures on cadavers and regularly in the patient care setting on MSK rotations over time.

A physician’s confidence in their ability is important, as they will be more likely to perform a MSK procedure if they feel confident in their skills. Our results showed that self-confidence was higher in those with previous experience and improved in both groups after training, with the highest self-confidence scores seen in the intervention group after both formal instruction and simulation training. Self-confidence in MSK procedures will allow primary care physicians to save patient time and cost of care, while decreasing unnecessary referrals and diagnostic imaging. However, this must not be false self-confidence at the expense of accuracy. One educational study evaluated proficiency of glenohumeral joint, subacromial space, lateral epicondyle, carpal tunnel, and knee injections performed by general practice trainees before and after an educational intervention involving lectures, cadavers, and anatomical models. Self-confidence concerning knowledge and skills was assessed before training, after training, and 3 months later. Three groups were created with modifications in each training style by lecture, training on anatomic models, or training on cadavers. Although all groups improved in knowledge and skill, one group reported higher self-confidence despite worse performance on the Objective Structured Clinical Examination (OSCE) [20]. It is important to ensure that self-confidence and accuracy align in order to achieve the best result. In our study, self-confidence scores correlated with improved injection accuracy. It is important to acknowledge that cadaver specimens are often expensive for an institution to purchase and store and it may not be feasible to obtain substantial materials for primary care physicians to practice. Prosthetic models are used for training in certain settings to bypass the logistics behind cadaver specimen management. One study demonstrated significant increases in comfort level amongst physicians when injecting after a workshop that involved pre-wired injection models of shoulders, wrists, hand, knee, and ankles [21]. The studies on injection models for both land-mark based and ultrasound-based demonstrate a clear and predictable benefit with practice and comfort level [21, 22]. However, there is limited literature on the benefits on accuracy from practice on these prosthetic models. Future research should involve comparing both confidence level and accuracy level between physicians with the opportunity to train on cadavers versus physicians with the opportunity to train on prosthetic models as well as a cost analysis between obtaining and maintaining cadaver specimens vs. models at an institution.

There are limitations to consider in this study. The subacromial space was chosen to examine proficiency due to its size and clinical relevance. However, results may be different if performed in another joint. Additionally, there was no power calculation because the study was limited by the number of cadavers (twelve) and it is possible that infiltrating the subacromial space multiple times may have affected the perceived accuracy of colored latex solution injectate. The technique scoring rubric may serve as a subjective evaluation, while the dissections are more objective. Therefore, it would be helpful to correlate these findings in the future possibly by having additional simultaneous evaluators for the rubric portion to evaluate the safety and sterility points of emphasis. Also, although participants were grouped randomly, their level of training and previous experience was varied, which may have confounded some of the results. As a result, different levels of experience may have confounded some of the pre-curriculum and post-curriculum results. However, our results showed that number of previous injections performed did not affect accuracy. Finally, there was no long-term data on participants’ improvement in the SAI in the clinical setting. The authors believe that this would be worth exploring in future studies.

Despite these limitations, the investigation provides an analysis of SAI accuracy and self-confidence before and after two types of education, with a clear difference promoting the use of integrated clinical instruction and simulation training over lecture-based learning and observation. This may serve as a guide for improving MSK care and efficiency amongst physicians to save time and unnecessary cost and diagnostic examinations prior to orthopedic specialist referral. A longitudinal component was not incorporated into this study, but the authors believe it would be valuable to build upon the study by involving more integrated integration and stratifying proficiency before and after dedicated MSK rotations in order to assess maintenance, improvement, or attrition of skills over time.

## Conclusions

Simulation and integrated instruction can significantly improve accuracy and confidence in procedures such as SAIs. Although primary care physicians and surgeons develop experience with MSK procedures in practice, this study demonstrates a role for improved early integrated instruction and simulation to improve accuracy and confidence. This may ultimately decrease costs and avoid unnecessary orthopedic surgery referrals, diagnostic tests, and earlier than desired surgical intervention.

## Data Availability

Additional images of the dissections as well as calculations for the data analysis may be made available upon reasonable request from the corresponding author.

## Abbreviations

MSK: Musculoskeletal
SAI: Subacromial Injection
PGY: Post-Graduate Year
OSCE: Objective Structured Clinical Examination
